# Determination of thyroid volume in infants with suspected congenital hypothyroidism—the limitations of both subjective and objective evaluation

**DOI:** 10.1259/bjro.20200001

**Published:** 2020-06-10

**Authors:** Chourouk Mansour, Yasmine Ouarezki, Jeremy Huw Jones, Morag Green, Emily Jane Stenhouse, Greg Irwin, Pia Hermanns, Joachim Pohlenz, Malcolm David Cairns Donaldson

**Affiliations:** 1 Lalla Meriem Provincial Hospital of Larache, Larache, Morocco; 2 Hassen-Badi Public Health Establishment, El-Harrach, Algiers, Algeria; 3 NHS Greater Glasgow and Clyde, Royal Hospital for Children, Queen Elizabeth University Hospital, Govan Road, Glasgow, G51 4TF, United Kingdom; 4 NHS Greater Glasgow and Clyde, Department of Radiology, Royal Hospital for Children, Queen Elizabeth University Hospital, Govan Road, Glasgow, G51 4TF, United Kingdom; 5 Children's Hospital, University Medical Center, Johannes-Gutenberg-University, Mainz, Germany; 6 University of Glasgow School of Medicine, Section of Child Health, Royal Hospital for Children, Queen Elizabeth University Hospital, Govan Road, Glasgow, G51 4TF, United Kingdom

## Abstract

**Objective:**

To compare two methods of assessing gland size on thyroid ultrasound in newborn infants with suspected congenital hypothyroidism (CH).

**Methods:**

Images from infants with eutopic glands referred between 2007 and 2013 were evaluated blind by two sets of observers. Subjective gland size was categorised as small, borderline-small, normal, borderline-large and large. Objective gland volume, calculated as the sum of each lobe using the prolate ellipsoid formula (length x width x depth x π/6), was put into corresponding categories: <0.8, 0.81–1.0, 1.1– <2.2, 2.2–2.4 and >2.4 ml, derived from normative Scottish data.

**Results:**

Of 36 infants, permanent CH was present in 17, transient CH in 17, status uncertain in 2. Mean (SD) intraobserver error for thyroid volume measurement was 0.11 (0.23) ml [8.3%]. Subjective assessment by two observers was discordant in only four (10.8%) infants. However, subjective *vs* objective evaluation was discordant in 14 (39%). Eight (three permanent, five transient CH) had large glands subjectively but normal glands objectively; and six (four transient CH) had normal glands subjectively but small glands objectively. The former infants all showed a single flattened curve to the anterior thyroid margin, giving an impression of bulkiness. Gland shape was normal in the latter infants.

**Conclusion:**

Neither subjective nor objective evaluation predicts permanent *vs* transient CH. Altered gland shape may confound both methods, and undermine use of the conventional formula for measuring lobe volume.

**Advances in knowledge:**

Until more refined methods are available for assessing thyroid size, both subjective and objective evaluation are recommended in CH.

## Introduction

Congenital hypothyroidism (CH) has an incidence in the UK of about 1 in 3500 births^1^ and is an important cause of preventable mental handicap.^2^ Newborn screening by measuring capillary thyroid stimulating hormone (TSH) on heel prick testing has revolutionised the outlook for CH in developed countries but remains a challenge in resource-limited parts of the world.

CH can be classified according to aetiology and duration. Primary CH is traditionally categorised as thyroid dysgenesis and thyroid dyshormonogenesis.^3^ Thyroid dysgenesis comprises ectopia, where the gland has failed to migrate normally, athyreosis in which no thyroid tissue is identifiable, and hypoplasia in a normally positioned, or eutopic, gland. Thyroid dyshormonogenesis refers to a group of inherited enzyme or other protein defects, with a structurally intact and usually enlarged eutopic gland.

Permanent CH must be distinguished from transient TSH elevation, the latter being common in sick or preterm infants.^4^ However, it is now recognised that some types of dyshormonogenesis such as DUOX2 deficiency, a relatively common form in the UK,^5^ can result in both transient and permanent CH.^6^

Thyroid imaging is an integral part of the assessment of infants referred with TSH elevation. The European Society for Paediatric Endocrinology (ESPE) guidelines recommend that either thyroid ultrasound or scintigraphy, or both modalities, should be carried out in suspected cases.^7^ It is particularly important to identify a eutopic thyroid gland in suspected CH; work from Rabbiosi and colleagues showing that only a third of children re-evaluated at 3 years needed to continue with L-thyroxine (L-T4) therapy.^8^

Assessment of thyroid size, while inapplicable in athyreosis and of limited value in ectopia, is important in CH with a eutopic gland, helping to establish aetiology and focus subsequent investigation. A small eutopic gland demands molecular genetic analysis to rule out mutations in one of the candidate genes for thyroid dysgenesis: *PAX8*, TSH receptor and *NKX2.1*.^9^ An enlarged gland *in situ* evokes causes such as iodine deficiency or dyshormonogenesis.^10^

**Figure 1.**
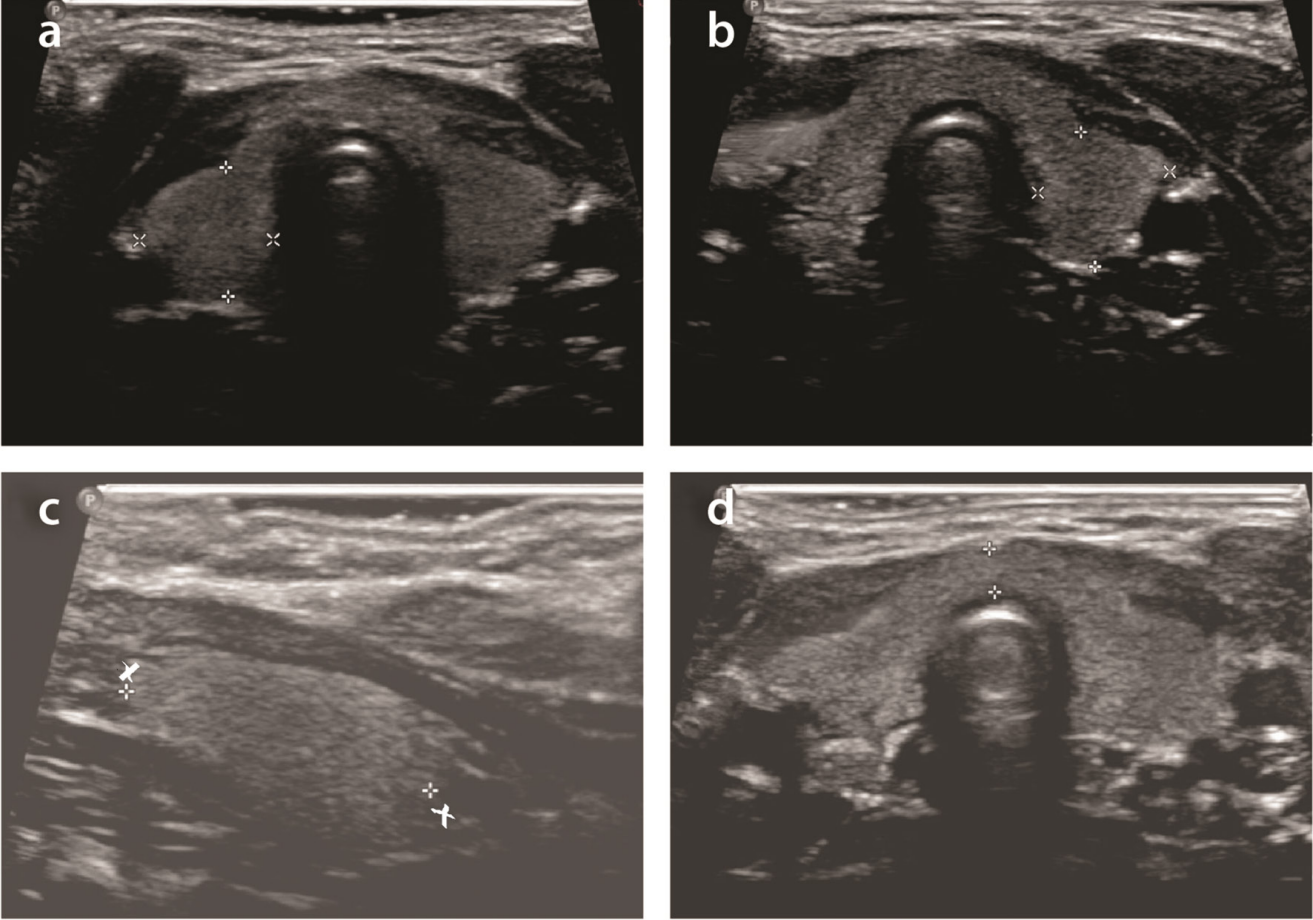
(a, b, c and d) Thyroid ultrasound images of normal infant showing width and anteroposterior measurement of right lobe (a), left lobe (b), length of right lobe (c) and anteroposterior measurement of the isthmus (d). Note that measurement of the thyroid isthmus is not factored into the formula for calculating thyroid volume.

Reference ranges for thyroid size in healthy newborn infants have been established in various countries, including Scotland.^11^ The length, width and anteroposterior dimension of each thyroid lobe are measured (Figure 1a, b and c) and volume calculated by assuming the shape of a prolate ellipsoid and applying the formula length x width x anteroposterior dimension x π/6 (or a similar constant). Thyroid volume is then expressed as the sum of both lobes. The contribution of the thyroid isthmus to volume (Figure 1d) is ignored.^11^

While formal measurement of thyroid volume may be recommended, subjective assessment of gland size by an experienced observer remains the norm in clinical practice. However, there are currently no data to validate either objective or subjective assessment of thyroid volume in pathological states, and no studies directly comparing the two methods.

**Figure 2.**
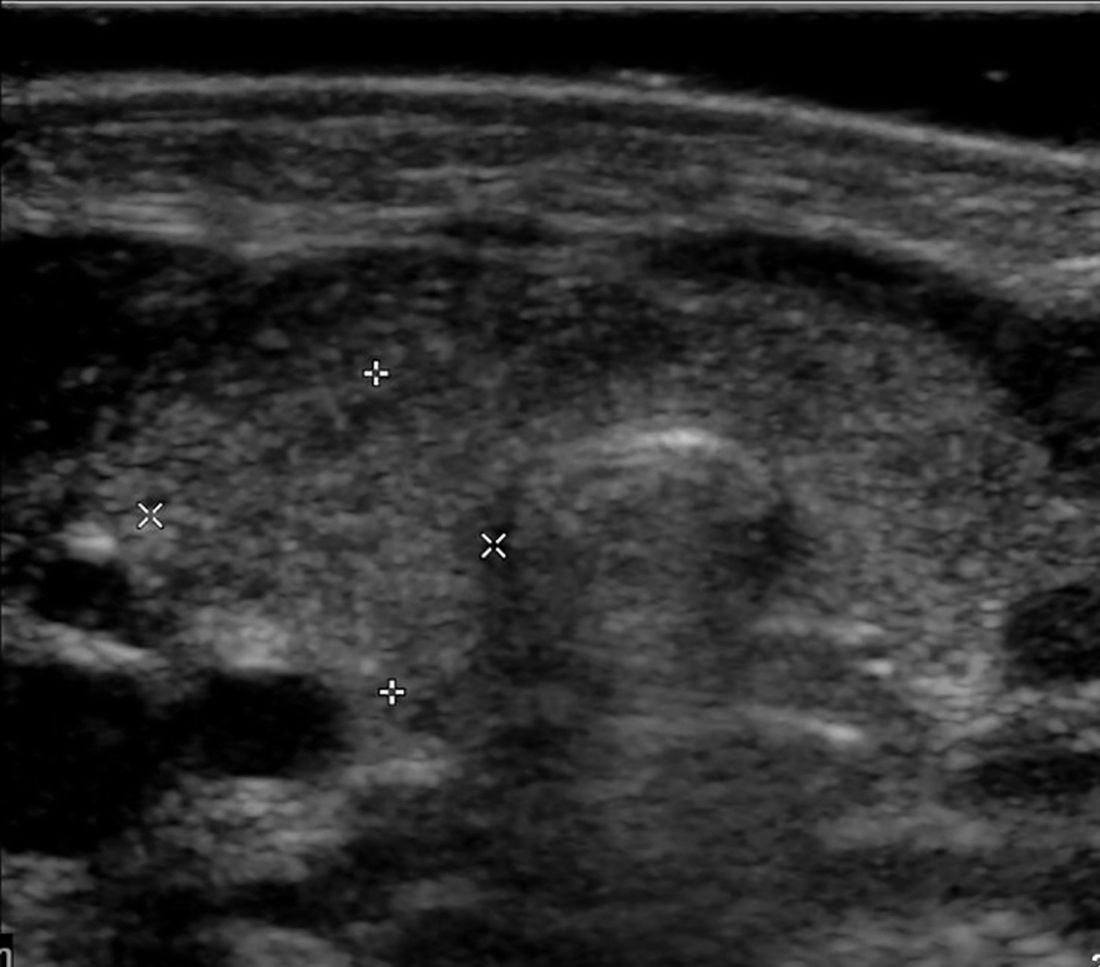
Thyroid ultrasound in an infant with Down syndrome and *PAX8* mutation (patient P11 in Table 1 and Hermanns et al). Thyroid appears bulky but combined lobe volume is actually small at 0.78 ml (reference range 0.8–2.4 ml).

We became aware of a potential discrepancy between subjective and objective thyroid assessment when an infant with Down syndrome in our centre was found to have a *PAX*8 mutation resulting in permanent CH.^12^ The infant’s thyroid gland had been subjectively reported on ultrasound as “bulky,” implying increased size (Figure 2). However, when the lobes were measured while preparing the case for publication, thyroid volume was found to be at the lower end of the reference range.

**Figure 7.**
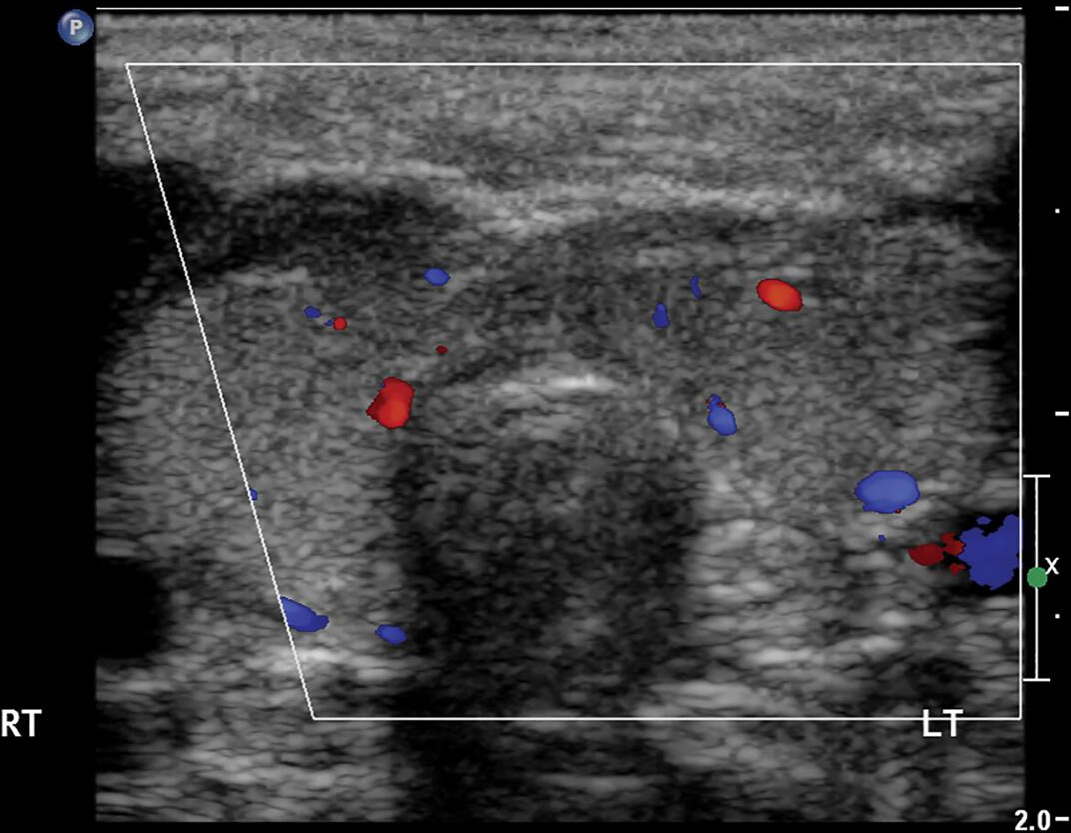
Ultrasound of infant with a variant of the brain–lung–thyroid syndrome with mutation adjacent to the *NKX2.1* gene (patient T6, Table 2). Subjective evaluation suggested a bulky gland attributable to a single, flattened, slightly curved anterior border but measured volume was small-normal at 0.94 ml. o PostScript

**Table 1.** Data on 17 infants with permanent (P) congenital hypothyroidism, ranked by measured thyroid size

No	Sex	BW (kg)	G (wk)	Mean Ox vol (ml)	Agreed Sx vol (ml)	RIS uptake	RIS size	Tg (μg/L)	Mutation found	Diagnosis	Comment
P1	M	4.02	42	4.7	Large	N/A	N/A	<2	Tg	DHG	Homozygote; affected sibling
P2	M	3.03	41	3.53	Large	↑	↑	1500	DUOX.2	DHG	Compound heterozygote
P3	M	1.66	35	3.43	Large	↑	↑	N/A	N/T	DHG	
P4	F	3.26	41	3.29	Large	↑	↑	<2	Tg	DHG	Compound heterozygote
P5	F	3.07	41	3.07	Large	↑	↑	>300	TPO	DHG	Compound heterozygote; affected sibling
P6	M	2.91	41	2.74	Large	N	↑	1562	TPO	DHG	Compound heterozygote
P7	F	3.26	40	1.75†	Large*	N	N	>300	DUOX.2	DHG	Compound heterozygote. Thick isthmus, increased vascularity (Figure 6)
P8	M	3.08	39	1.56†	Large*	N	↑	2087	No	DHG	Thickened isthmus, increased vascularity
P9	F	3.54	39	1.2	Normal	N	N	1503	No	U/K	Diagnosed as DHG prior to study
P10	M	3.43	41	0.8	Borderline-small	Absent	-	21	TSH-R	Hypoplasia	Heterozygote (mother affected); Mild TSH elevation but normal fT4 off treatment
P11	F	2.83	38	0.78†	Borderline-large*	↓	N	N/A	PAX8	Hypoplasia	Index case. Down syndrome with apparently ‘bulky’ thyroid on ultrasound(Figure 2) [ref 12]
P12	M	3.35	39	0.69†	Normal	N/A	N/A	N/A	N/T	Hypoplasia	Down syndrome. Hypothyroidism confirmed by diagnostic challenge at 5 years
P13	F	3.63	39	0.37	Small	↓	↓	219	No	Hypoplasia	Normal TSH-R testing
P14	M	4.96	40	0.35	Small	↓	↓	32	TSH-R	Hypoplasia	Two alleles affected; severe hypoplasia
P15	M	3.43	39	0.29	Small	↓	↓	N/A	TSH-R	Hypoplasia	Down syndrome with heterozygous TSH-R mutation
P16	F	1.92	37	0.23	Small	N	↓	N/A	N/T	Hypoplasia	Cause unknown
P 17	M	3.94	40	0.16	Small	↓	↓	43	PAX8	Hypoplasia	Severely hypoplastic gland

↓, decreased; ↑, increased; BW, birthweight; DHG, dyshormonogenesis; G, gestation; L-T4, levo-thyroxine; N, normal; N/D, not done; Ox, objective; Sx, subjective assessment; TPO, thyroid peroxidase; Tg, thyroglobulin.

*single curve to anterior border (see text)

†objective and subjective assessment discordant by two categories. Reference range for Tg 63–403 µg l^−1^, reference range for Ox volume 1–2.2 ml.

**Table 2.** Data on 17 infants with transient (T) congenital hypothyroidism and two in whom status remains uncertain (SU), ranked by measured thyroid size

No	Sex	BW (kg)	G (wk)	Mean Ox vol (ml)	Agreed Sx vol (ml)	RIS uptake	RIS size	Tg (μg/L)	Mutation found	Diagnosis	Comment
T1	F	2.85	40	2.42	Large	↑	N	2301	Pendrin	DHG	Heterozygote. Thickened isthmus, ↑ vascularity. Treated with L-T4 for 6 years
T2	F	2.63	40	2.0†	Large*	↑	N	1717	No	DHG	↑ vascularity Treated with L-T4 for 4 years
T3	M	2.86	37	1.80†	Large*	N	N	4995	No	DHG	Thickened isthmus, ↑ vascularity
T4	F	3.06	38	1.17	Normal	N/D	N/D	N/A	-	Sick	Placental abruption, birth asphyxia, renal failure.
T5	M	3.12	39	1.16†	Large*	N	N	>1000	No	DHG	Thickened isthmus, ↑ vascularity
T6	F	3.07	40	0.93†	Large*	N	↓	857	Yes	Hypoplasia	Brain-lung-thyroid syndrome with deletion proximal to *NKX2.1*.(Figure 7) [ref 18]
T7	M	3.80	39	0.85†	Large*	N	N	1810	No	U/K	DHG diagnosed initially. Treated for 3 years
T8	F	2.37	37	0.84	Normal	↓	↓	259	-	U/K	Never treated
T9	F	3.57	40	0.83	Normal	N/D	N/D	N/A	-	U/K	Treated during infancy
T10	F	2.82	37	0.76†	Normal	N	↓	N/A	-	Thyr-ab	Turner's syndrome (45,X). TPO antibodies 30.8 IU ml^−1^ in infant & 155.9 in mother. Not treated.
T11	M	3.42	40	0.75†	Normal	↓	↓	N/A	-	U/K	Never treated
T12	M	3.80	39	0.72	Borderline-small	↓	N	N/D	-	U/K	Treated for 3 years
T13	M	3.60	41	0.63†	Normal	Absent	-	N/A	-	Thyr-ab	Blocking maternal TSH receptor antibodies (21 u l^−1^). Sibling also affected.
T14	M	3.00	42	0.62	Normal	N/D	N/D	N/A	-	U/K	Treated briefly in infancy
T15	F	3.42	40	0.61†	Normal	↓	↓	344	TSH-R	Hypoplasia	Heterozygous TSH-R. Treated for 2 years.
T16	M	2.48	37	0.60	Borderline-small	↓	N	37	-	U/K	Never treated
T17	M	3.65	41	0.48	Borderline-small	Absent	-	50		Thyr-ab	TPO antibodies present in infant (73.6 IU ml^−1^).
SU1	M	3.18	38	1.28	Borderline-large*	↑	↑	N/A	-	U/K	Down syndrome; complex congenital heart disease.
SU2	M	1.44	31	0.5†	Normal	↓	↓	N/A	15q 11.2 deletion	U/K	Not retested yet in view of learning difficulties

BW, birthweight; DHG, dyshormonogenesis; G, gestation; L-T4, levo-thyroxine; N/D, not done; Ox, objective; Sx, subjective; TPO, thyroid peroxidase; Tg, thyroglobulin; d, decreased; i, increased.

*single curve to anterior border (see text)

†objective and subjective assessment discordant by two categories Reference range for Tg 63–403 µg l^−1^, reference range for Ox volume 1–2.2 ml.

This observation prompted the present study which aims to compare objective and subjective assessment of thyroid size in a group of infants with TSH elevation and eutopic glands in whom at least 6 years of follow up are available. The study also determines the intraobserver variability of objective measurement and the interobserver variability of subjective assessment.

## Patients and methods

Newborn screening for CH began in Scotland in 1979.^13^ In 1990, a database designed for gathering information and helping with follow up was established and has been maintained and audited since.^1,13,14^ From the outset, all infants referred on the programme have had venous blood taken to confirm TSH elevation (paediatric reference range 0.3–5.5 mU/L) and to gauge the severity of hypothyroidism by measuring free T4 (paediatric reference range 9–26 pmol l^−1^). An assay for serum thyroglobulin (Tg), a protein synthesised exclusively in the thyroid, has been available in Glasgow since 2004 using IMMULITE 2000 (Siemens), FS 2 µg l^−1^, reference range 63–403 µg l^−1^ based on Czech data from 26 healthy singleton infants (Neumann *et al*, 2020 in preparation). Since then, Tg measurement has been carried out by some but not all centres, at the clinician’s discretion.

Thyroid imaging in suspected CH has been practised throughout Scotland with varying consistency since screening began. Since 1999, a comprehensive service offering both thyroid ultrasound and radioisotope scanning has been established and developed in the Royal Hospital for Sick Children in Glasgow^15,16^ . Of the 17–41 (median 28) referrals per year in Scotland between 1999 and 2019, dual scanning has been offered to infants from the West of Scotland and extended to other Scottish Health Boards on request.

In the present study, thyroid ultrasound images of all newborn infants with TSH elevation referred to our centre for imaging between 2007 and 2013, in whom a thyroid gland *in situ* (eutopic gland) had been identified, were reviewed using the Picture Archiving and Communications System (PACS), (Kodak Carestream, Carestream Health, Station Road, Hemel Hempstead, UK). Patients in whom good quality images of the entire thyroid gland were available were included.

Molecular genetic analysis had been carried out in selected cases at the clinicians’ discretion in Glasgow, Scotland, and Mainz, Germany. Genes analysed included those encoding the thyroid stimulating hormone receptor (TSH-R), Tg, Pendrin, the transcription factor PAX8, and the enzymes thyroid peroxidase (TPO), DUOX.2 and DUOX.A2.

Ultrasound imaging was carried out using a Philips iU22 model (Koninklijke Philips Electronics N.V., Groenewoudsweg 1, 5621 BA, Eindhoven, The Netherlands) featuring a 7–15 MHz hockeystick transducer. Radioisotope imaging used ^99m^Tc Pertechnetate with a high-resolution pin hole collimator (either Siemens Symbia, Siemens Medical Solutions, 2501 North Barrington Road, Hoffman Estates, IL or Phillips Axis, Phillips Medical Systems, The Observatory, Castlefield Road, Reigate, Surrey, UK). When, despite using the widest field of view available, it was not possible to include the whole thyroid lobe, length was determined by extrapolation,

### Evaluation of thyroid images

Two observers (MG and JJ), who were blinded to clinical details or previous reports, recorded the length, width and anteroposterior dimension of each infant’s lobes on two separate occasions. Lobe volume was calculated as: (length x width x anteroposterior dimension x π/6)/1000 to give a value in millilitres and combined thyroid volume expressed as the sum of both lobes as previously described.^17^ In the present study, glands were classified as small, borderline-small, normal, borderline-large and large corresponding to volumes <0.8, 0.8–1.0, 1.01–< 2.2, 2.2–2.4 and >2.4 ml, based on the normative Scottish data of Perry et al in which mean (SD) thyroid volume was 1.6 (0.4) ml.^11^

As a separate exercise, a radiographer and radiologist (MG and ES) independently assessed the ultrasound images subjectively, under blinded conditions, scoring glands as large, borderline-large, normal, borderline-small, or small. These observers then discussed their findings to reach an agreed score in each patient.

Finally, the available radioisotope (RIS) images were evaluated under blinded conditions on two occasions by a single radiologist (GI) and scored as showing absent, decreased, normal or increased uptake; and small, normal or increased volume. GI then reviewed all images with ES during a second session to reach a final evaluation.

In 2019, the thyroid status in each patient was revisited using Clinical Portal (https://www.ggc-clinicalportal.scot.nhs.uk/concerto) and where necessary by contacting the relevant paediatrician.

### Diagnostic classification of patients

A modification of the double-classification for CH previously described^1^ was applied, according to: (a) duration; and (b) aetiology.

#### Duration

CH was categorised as ‘permanent’ in patients who, by 2019 were **either** still receiving L-T4 therapy **or** (in one case) who had persistent TSH elevation off treatment.

‘Transient’ CH was defined when normal venous free T4 and TSH values were recorded, either in patients in whom treatment had not been required, or after L-T4 had been withdrawn as a diagnostic challenge.

Patients in whom the cause of CH had not been established and who remained on L-T4 pending diagnostic challenge were categorised as ‘Status uncertain‘.

#### Etiology

The term ‘dyshormonogenesis’ was applied to patients with either permanent or transient CH in whom there was **either** a molecular genetic diagnosis (*e.g.* TPO, Tg, DUOX.2 defect) **or** agreed gland size was increased **or** gland size was normal in the presence of at least **three** additional features consistent with dyshormonogenesis, comprising: increased size of gland on RIS, increased uptake on RIS, enlargement of the thyroid isthmus and increased vascularity on thyroid ultrasound; and markedly elevated Tg (>1000 µg l^−1^) at presentation.

Thyroid hypoplasia was defined as **either** permanent CH in a patient in whom gland size was agreed to be small on combined subjective or objective evaluation; **or** an objectively small or borderline-small gland in a patient with a gene disorder known to be associated with reduced thyroid volume such as *PAX8* and *TSH-R* mutation.

The term ‘permanent CH of unknown cause’ was applied to patients in whom agreed gland size was objectively normal rather than small or large in a patient shown to be persistently hypothyroid on diagnostic challenge in the absence of features suggestive of dyshormonogenesis.

#### Statistical aspects

Intraobserver differences were calculated between the first and second measurement of combined thyroid volume for all infants. Correlation between the objective and subjective assessment of thyroid size was described as concordant; partially concordant if one category apart; and discordant if two categories apart.

#### Ethical aspects

All parents had given informed consent at the time of assessment for the images to be stored and later assessed in an anonymised form. The study was registered with the Clinical Effectiveness Department at NHS Greater Glasgow & Clyde as a Quality Improvement Project. In 2019, Caldicott approval was obtained from the Guardian for NHS Greater Glasgow and Clyde to access patients on Clinical Portal, the Caldicott Guardian being a senior person in a given Health Board who is responsible for protecting the confidentiality of patient health and care information and ensuring that patient anonymity is maintained.

## Results

**Figure 3.**
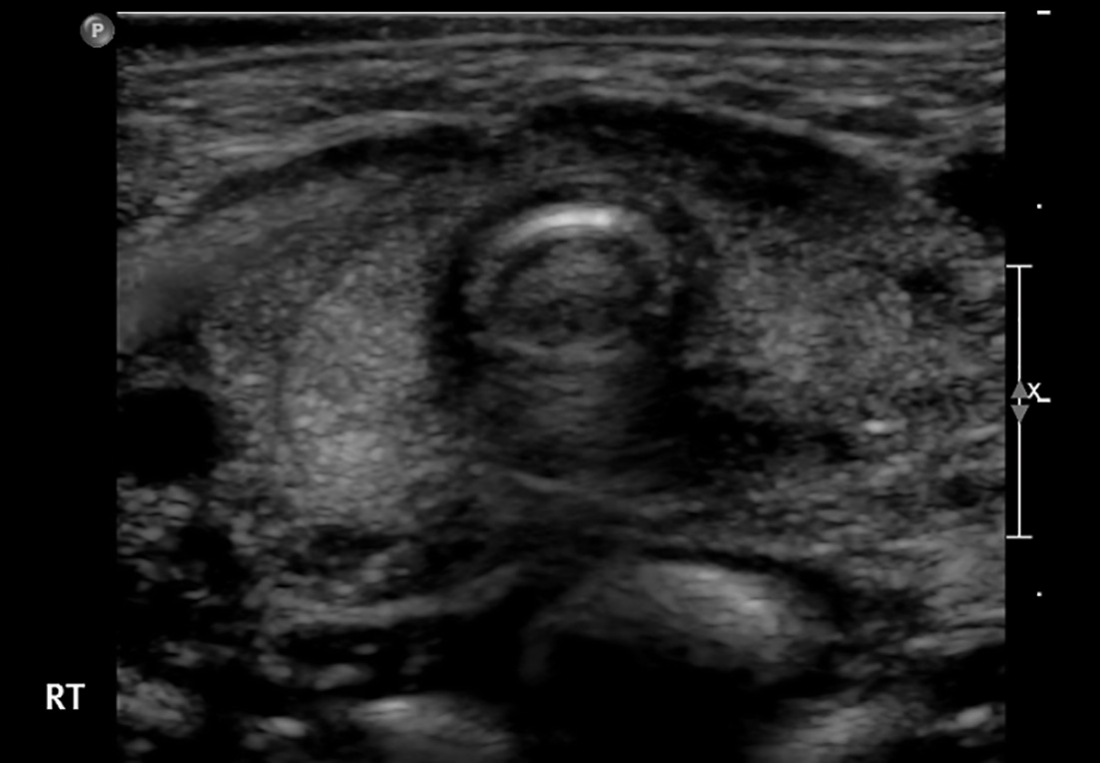
Ultrasound image showing perithyroid oedema due to allo-immune thyroiditis in an infant born to a mother with very elevated TSH-receptor antibodies (>405 u l^−1^). Neither subjective evaluation nor objective measurement was possible in this patient.

Stored images permitted measurement of both lobes in 37 of 61 infants with TSH elevation who were recorded as having a eutopic gland during the study period. One infant with allo-immune thyroiditis from maternal antibodies and transient congenital hypothyroidism was excluded, since perithyroid oedema confounded both objective and subjective assessment in this patient (Figure 3).

Of the remaining 36 patients (21 males, 15 females) median (range) birthweight, gestation and age at thyroid ultrasound examination were 3220 (1440–4460) g, 39.5 (31–42) weeks and 19 (7-62) days, respectively. Five infants were of low birth weight (<2500 g) and one preterm (<37 weeks) at 31 weeks gestation.

### Categorisation and diagnostic classification (Tables 1 and 2)

Of the 36 study patients, 17 have permanent CH (P1-17) and their data are shown in Table 1. Table 2 gives details on 17 patients with transient CH (T1-17), and in 2 (SU1-2) in whom status remains uncertain.

12 patients met the criteria for dyshormonogenesis of whom eight had permanent and four had transient CH, with molecular genetic confirmation in seven comprising *TPO* (2), *Tg* (2), *DUOX.2* (2) defect and *Pendrin* heterozygote (1).

The criteria for thyroid hypoplasia were met in 10 patients, eight with permanent and two with transient CH. *TSH-R* mutation was found in four patients, homozygous in one (P14) and heterozygous in three (P10, P15, T15), one of whom (P15) has Down syndrome. Of note, radioisotope uptake was decreased or absent in all four patients. *PAX8* mutation was found in two patients (P11, P17), including the index patient (P11) with Down syndrome.^12^ A further patient (T6), presented with TSH elevation on newborn screening, commenced L-T4 therapy, but then developed neuromotor symptoms and was found to have a deletion proximal to the *NKX2.1* gene, consistent with a variant of the brain–lung–thyroid syndrome.^18^ This patient’s thyroid function became normal off treatment at 4 years of age. Cause in the remaining three patients with thyroid hypoplasia (P12,13 and 16), one of whom (P12) has Down syndrome, is unknown.

Amongst the nine patients with transient CH who were not categorised as having dyshormonogenesis or hypoplasia, cause was attributable to thyroid autoantibodies in three (patients T10,13,17), two of whom showed absent uptake on RIS; and to perinatal asphyxia in one (patient T4). Cause was not established in the remaining seven patients.

Diagnostic challenge has not yet been carried out in two patients, related to ongoing problems—complex cardiac disease and Down syndrome (SU1), and neurodisability related to a 15q deletion (SU2).

#### Intraobserver error for objective measurement of thyroid ultrasound volume (Table 3)

**Table 3.** Intraobserver error for blind evaluation of measured thyroid volume on ultrasound by the same two observers on two separate occasions in 36 newborn infants with elevated thyroid stimulating hormone levels on newborn screening, and eutopic glands

Gland volume [n=]	Mean volume [n=]	Intra observer difference		Mean % difference
		Mean ± SD	Median	
Small (<0.8 ml)	0.55 [*n* = 17]	0.049 ± 0.035	0.0421	4.9
Borderline-small (0.8–1.0 ml)	0.86 [*n* = 4]	0.047 ± 0.05	0.0341	4.7
Normal (1.01–2.19 ml) all patients	1.49 [*n* = 8]	0.0812 ± 0.077	0.0527	8.1
Normal (1.01–2.19 ml) all patients, excluding infants in whom extrapolation was needed	1.40 [*n* = 6]	0.0969 ± 0.083	0.085	9.6
Borderline-large (2.2–2.4)	[*n* = 0]			
Large (>2.4 ml all patients	3.30 [*n* = 7]	0.3755 ± 0.45	0.1295	37.5
Large (>2.4 ml) excluding infants in whom extrapolation needed	3.08 [*n* = 5]	0.1579 ± 0.1609	0.1043	15.7

Mean gland volumes between sessions 1 and 2 were grouped according to size categories derived from the data of Perry et al.^11^

Mean ± SD (%) differences in right lobe volume, left lobe volume and combined volumes between the first and second sessions were 0.07 ± 0.13 (9.7%), 0.06 ± 0.10 (10%) and 0.11 ± 0.23 (8.3%) respectively.

**Figure 4.**
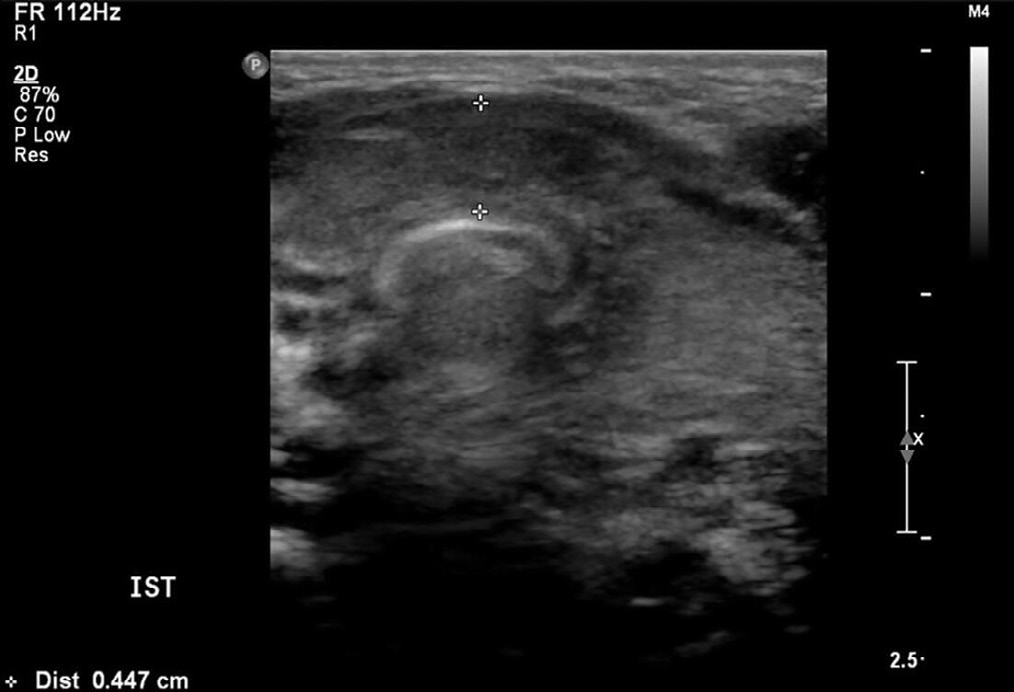
Enlarged thyroid in an infant with dyshormonogenesis due to a thyroid peroxidase gene defect (patient P6 in Table 1). The thyroid lobe is too large to be shown on one image so that its length had to be estimated by extrapolation.

Intraobserver difference was greater—0.38 ml (37.5%)—in the seven infants with large glands. This difference fell to 0.16 ml (15.7%) after excluding two patients in whom thyroid length had to be estimated by extrapolation, the lobes being too large to be viewed on a single image (Figure 4).

### Interobserver error for subjective measurement of thyroid ultrasound volume

The two observers were concordant for 24, one category apart for 8 and discordant for 4. These latter patients comprised borderline-large *vs* small (agreed borderline-large) in the index patient with *PAX8* mutation (patient P11, Figure 2); and small *vs* normal (agreed borderline-normal) in three.

### Concordance between objective and subjective evaluation of thyroid volume (see Figure 5, Tables 1&2)

**Figure 5.**
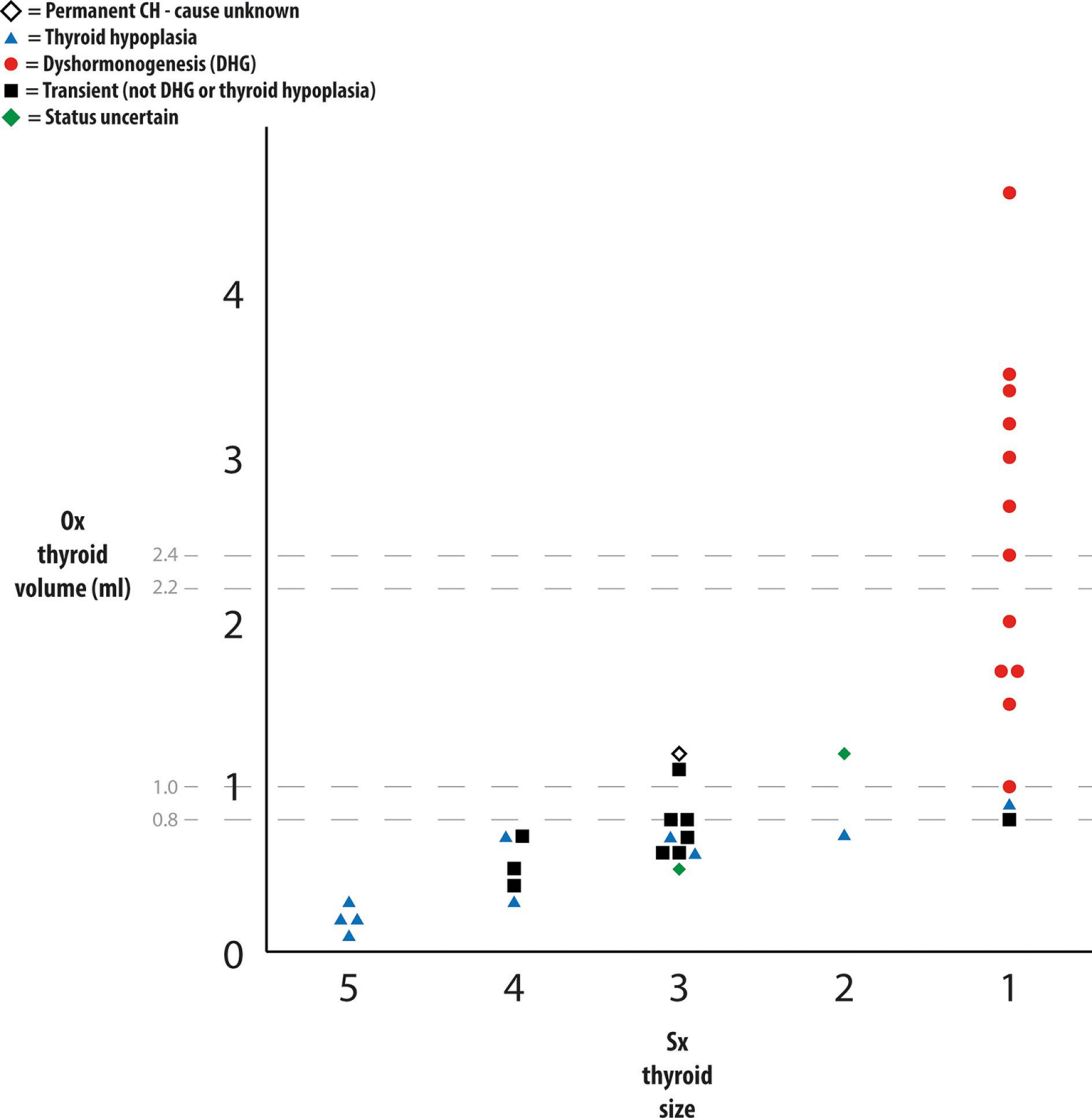
Chart comparing subjective evaluation with objective assessment of thyroid size in 36 infants referred with elevated thyroid stimulating hormone on newborn screening. Subjective size (small, small-normal, normal, large-normal and large) is shown on the x-axis and objective measurement of thyroid volume is shown on the y-axis. Individual patients are colour-coded according to diagnostic category.

Figure 5 shows the comparison between subjective and objective evaluation of thyroid size related to the final diagnostic category in 2019. The two modalities were concordant (14) or one category apart (8) for 22 patients. However, subjective and objective evaluation was discordant (two categories apart) for 14 patients—P7,8,11,12; T2,3,5,6,7,10,11,13,15; and SU2 (denoted with a † symbol in Tables 1 and 2).

In eight patients, thyroid size was assessed as large (7) or borderline-large (1) on subjective evaluation but objectively of normal (5), borderline-small (2) or small (1) volume. In all these infants (denoted with a * symbol in Tables 1 and 2), thyroid shape was altered, with the normal curves of the anterior margin of the thyroid gland (Figure 1) being replaced by a single curve (Figures 2 6 and 7 corresponding to patients P11, P7 and T6) giving a bowed appearance and hence an impression of enlargement or bulkiness. Moreover, the thyroid isthmus was judged to be enlarged in four of these patients (P7,P8, T1 and T3).

**Figure 6.**
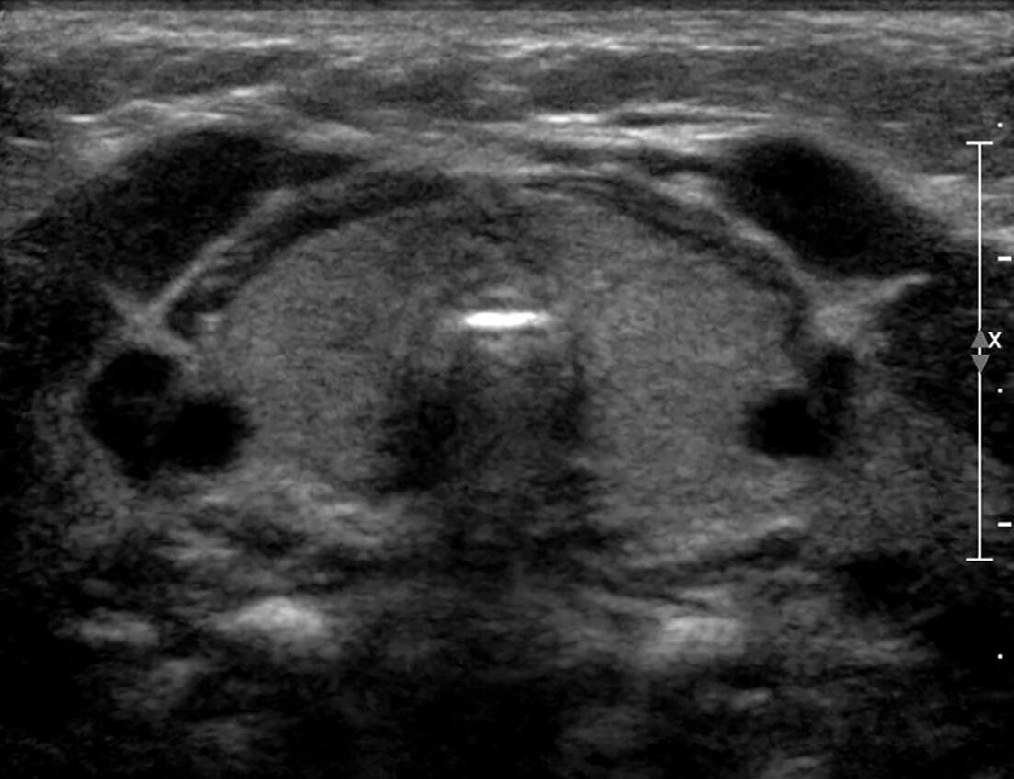
Ultrasound of infant with compound heterozygous *DUOX.2* mutation (patient P7, Table 1), showing loss of normal curves of anterior margin of thyroid. Objective measurement of the gland at 1.75 ml falls within normal limits.

In the remaining six infants with discordance between objective and subjective assessment (patient P12, Table 1; patients T10,11,15 and SU2 in Table 2), the gland was subjectively normal but objectively small (<0.8 ml) on measurement. Gland shape was normal in all these patients in whom measured thyroid volume ranged between 0.52 and 0.76 ml.

## Discussion

This study both confirms the importance of carrying out thyroid imaging in suspected CH and highlights the current limitations of assessing gland size with thyroid ultrasound according to conventional methods.

Diagnostic imaging, by identifying the presence/absence and location of thyroid tissue, is essential for the management and counselling in babies with TSH elevation on newborn screening. Thyroid ultrasound is reliable for detecting a gland *in situ*, provided that the diagnostic trap of misinterpreting non-thyroidal fatty tissue in the thyroid fossa as thyroid dysplasia is avoided.^19^ However, radioisotope imaging is required if thyroid ectopia is to be securely identified.^15,16^

If athyreosis or ectopia is demonstrated, lifetime treatment with L-T4 will be required. However, if a eutopic gland is identified then there is a significant chance of transient thyroid dysfunction—34.5% of patients in an Italian study of 86 children,^8^ and 17 of the 36 patients (45.9%) in this study. Thus, while the finding of a eutopic gland encourages the clinician to seek a specific diagnosis (*e.g.* gene mutation) where possible, it mandates critical evaluation of the diagnosis of permanent CH, with diagnostic challenge at 3 years in selected cases, as recommended by current European guidelines.^7^ It is therefore to be hoped that diagnostic imaging, whether by ultrasound, isotope scanning or both, will be more consistently performed throughout Scotland and the rest of the United Kingdom in the future.

To date, studies and guidelines involving ultrasound assessment of eutopic thyroids have used terms such as ‘normal’ or ‘normally shaped’, ‘enlarged’ or ‘goitre’, and ‘hypoplastic’ or ‘morphologically abnormal’ without the systematic use of standards and cut-offs,^7,10,20,21^ relying on the interpretation of an experienced observer.^10,21^ This study challenges and critically examines this aspect of diagnostic imaging.

Concerning the diagnosis of dyshormonogenesis, our data show that this should not depend solely on measured thyroid volume which was well within the reference range at 1.75 ml in one of our patients with proven *DUOX.2* mutation (patient P7, Table 1, Figure 6). Even if it were possible to measure thyroid volume accurately in CH infants with altered gland shape, applying rigid cut-offs would be unlikely to result in 100% sensitivity. We believe that it more appropriate to take account of additional factors such as uptake and apparent size on RIS, increased vascularity on ultrasound, and thyroglobulin levels, which are usually greatly elevated except in cases of *Tg* mutation (see patients P1and P4 in Table 1). These factors, rather than size alone, are reflected in the criteria we have set for diagnosing dyshormonogenesis in the present study.

Equally, gland size cannot be used as the sole criterion for thyroid hypoplasia. A small thyroid gland may be normal, particularly in a small infant with transient TSH elevation. For this reason, we have defined thyroid hypoplasia strictly, as small or borderline-small gland volume in either permanent CH or in the presence of a gene disorder known to result in impaired thyroid size.

While the criteria and standards set in our study may be open to question and debate, they at least represent an attempt to bring rigour to an area of paediatric thyroid disease which has not, to date, received critical attention.

Intraobserver error for objective measurement and interobserver error for subjective assessment in this study are favourable. When thyroid volume was derived using a standard formula the data were reproducible, with an intraobserver error of less than 10%. Accuracy was undermined when the gland was too large for one lobe to be visualised in a single field so that extrapolation was necessary (Figure 4), a problem highlighted by Ueda et al, who recommended measurement of lobe width and anteroposterior dimension/thickness but not length in neonates.^22^ While a larger footprint linear transducer would allow a wider field, this technique is not feasible in newborn infants, in whom the neck area is too small to access.

Subjective assessment of gland size was also reasonable in this study, the observers independently scoring either the same or only one category apart for 32 of the 36 (86.5%) cases.

By contrast, our study demonstrates a major problem of discordance between subjective and objective assessment, affecting a significant number (14/36) of cases. This discordance concerns: (a) subjectively large glands scoring normal or even small for size on objective measurement; and (b) subjectively normal-sized glands scoring small on objective measurement.

In the former category, the principal factors causing discordance were thyroid shape and size of isthmus. When the normal curves of the anterior margin of the thyroid gland (Figure 1) are replaced by a single curve, giving a bowed appearance (Figure 2), the presence of enlargement or bulkiness is suggested. This led to an erroneous subjective assessment in glands which were initially judged to be enlarged but were in fact mildly hypoplastic in association with *PAX8* mutation, and mutation adjacent to the *NKX2.1* gene (patient P11, Figure 2, patient T6, Figure 7).

However, objective assessment using standard methodology may also be confounded by altered gland shape, since the formula which assumes the shape of a prolate ellipsoid in each lobe may no longer apply. Also, the size of the isthmus, which is typically enlarged in cases of dyshormonogenesis (Figure 4), is not factored into the formula for thyroid volume measurement.

Of note, there were six cases where subjective evaluation indicated a normally shaped thyroid gland of normal size but in whom measured volume was up to 0.2 ml below our defined limit of 0.8 ml. Moreover, 7 of the 17 cases of transient CH showed measured volumes of <1 ml but normal size and normal shape on subjective examination (see patients T9-11, 13–15, Table 2). This discrepancy might be partly accounted for by the relatively high reference range established for Scottish data. Certainly, thyroid size was greater in Perry’s study than in comparable studies from countries such as Turkey,^23^ Poland,^24^ China,^25^ Germany^26^ and Belgium^27^ (Table 4). Smoking has been shown to have a goitrogenic effect in adults, with increased thyroid size in the newborn of mothers who were smokers^28^ and neither smoking nor iodine status were evaluated in Perry’s study.^11^ Iodine status in Scottish mothers is currently the subject of a large clinical study and preliminary results suggest that fewer than 50% of a pregnant females sample population satisfy the World Health Organisation criteria of urinary iodine concentration ≥150 µg l^−1^ for iodine sufficiency^29^ (Combet, 2020 in preparation). Nevertheless, even according to Polish and German data, the thyroid volumes in 11 of our transient cases are at the lower end of the reference range. It is possible, therefore, that some causes of transient CH, such as anti-thyroid antibodies may reduce gland size. This finding has implications for the follow up of infants with transient TSH elevation, who are currently discharged once thyroid function has normalised.

**Table 4.** Summary of normative thyroid ultrasound data in newborn infants (birth − 28 days) for thyroid lobe length, width, anteroposterior dimension and combined volume of both lobes in studies from Scotland, Poland, Turkey, China, Germany and Belgium

	RIGHT LOBE				LEFT LOBE				BOTH LOBES
	Length (cm)	Width (cm)	Anteroposterior (cm)	Vol (ml)	Length (cm)	Width (cm)	Anteroposterior (cm)	Vol (ml)	Vol (ml)
**Scotland, Perry et al 2002 (100 newborn) [π/6]. *See reference ^11^***									
Mean (SD)	1.94 (0.24)	0.87 (0.15)	0.97 (0.16)	0.81 (0.23)	1.94 (0.24)	0.89 (0.16)	0.95 (0.17)	0.82 (0.24)	1.62 (0.41)
Range	(0.9–2.5)	(0.5–1.4)	(0.6–2.0)	(0.3–1.7)	0.9–2.4)	(0.6–1.4)	(0.7–1.9)	0.4–1.7)	(0.7–3.3)
**Poland, Mikolajczak et al 2014 (148 newborn) [π/6] *See reference^24^***									
Mean (SD)	2.05 (0.15)	0.71 (0.1)	0.68 (0.075)	0.53 (0.1)	2.0 (0.155)	0.73 (0.104)	0.68 (0.07)	0.52 (0.13)	1.05 (0.24)
	(1.5–2.9)	(0.5–1.0)	(0.5–0.9)	(0.2–0.9)	(1.6–2.7)	(0.5–1.0)	(0.5–1.0)	(0.3–1.0)	(0.5–1.8)
**Turkey, Kurtoglu et al. 2008 (100 term newborn) [0.479] *See reference^23^***									
Mean									0.72 (0.9)
Range									
**China, Yao et al. 2010 (85 newborn) [0.479] *See reference^25^***									
Mean	1.46 (0.27)	0.72 (0.14)	0.63 (0.11)		1.45 (0.23)	0.72 (0.13)	0.61 (0.13)		0.64 (0.27)
Range	(0.7–2.1)	(0.5–1.4)	(0.44–0.99)		0.72–1.9	0.45–1.13	0.39–1.1		(0.27–1.85)
**Germany, Klingmüller et al. 1992 (24 newborn) [π/6] *See reference^26^***									
Mean (SD)									1.1 (0.7)
Range									0.4–3.5
**Belgium, Chanoine et al 1991 (85 newborns) [π/6] *See reference^27^***									
Mean (SD)									0.83 (0.38)
Range									

The constant used to calculate thyroid volume is given as either π/6 (0.52) or 0.479.

Improved methods are clearly needed for assessing thyroid size in suspected CH. At present, no reference data exist for thyroid isthmus thickness in healthy newborn infants. Also, existing reference data are for healthy, term neonates whereas some of the infants referred with TSH elevation are preterm and hence small. Establishing a ratio between lobe length and tracheal width could be helpful in factoring in birth size. Therefore, further normative population data which examines not only lobe volume, but also isthmus thickness and tracheal width, and which takes account of maternal smoking and iodine status, is recommended

Another approach would be to develop techniques for assessing total thyroid volume, rather than lobe size. Ying et al recommend performing a three-dimensional scan,^30^ but this approach is not always readily accessible, has not been validated in the newborn, and will not help in deciding what equation to apply. Tracing around the gland on a single transverse image may be more accurate than assuming a standard shape and Shabana et al have suggested that interobserver variation could be reduced by applying this method.^31^

We conclude that both objective and subjective measurement of a eutopic thyroid gland are necessary; that neither method is entirely satisfactory due to the confounding effects of altered thyroid shape; and that a clear distinction between thyroid hypoplasia, normal size and thyroid enlargement are not always possible with ultrasound. Until better models for assessing thyroid size are available, we recommend using both subjective and objective assessment in evaluating thyroid volume. Our experience confirms that combining RIS with thyroid ultrasound is valuable.^15,16^ Finally, this study demonstrates the value of securing a precise molecular genetic diagnosis where possible, and the importance of long-term follow-up.
